# Development of a screener to assess athlete risk behavior of not using third-party tested nutritional supplements

**DOI:** 10.3389/fnut.2024.1381731

**Published:** 2024-05-15

**Authors:** Floris C. Wardenaar, Kinta D. Schott, Ryan G. N. Seltzer, Christopher D. Gardner

**Affiliations:** ^1^College of Health Solutions, Arizona State University, Phoenix, AZ, United States; ^2^Department of Medicine, Stanford University, Palo Alto, CA, United States

**Keywords:** batch testing, dietary supplements, sport foods, ergogenic aids, doping

## Abstract

**Introduction:**

The aim of this cross-sectional study was to develop an algorithm to predict athletes use of third-party tested (TPT) supplements. Therefore, a nutritional supplement questionnaire was used with a section about self-reported TPT supplement use.

**Methods:**

Outcomes were randomly assigned to a training dataset to identify predictors using logistic regression models, or a cross-validation dataset. Training data were used to develop an algorithm with a score from 0 to 100 predicting use or non-use of TPT nutritional supplements.

**Results:**

A total of *n* = 410 NCAA Division I student-athletes (age: 21.4 ± 1.6 years, 53% female, from >20 sports) were included. Then *n* = 320 were randomly selected, of which 34% (*n* = 109) of users consistently reported that all supplements they used were TPT. Analyses resulted in a 10-item algorithm associated with use or non-use of TPT. Risk quadrants provided the best fit for classifying low vs. high risk toward inconsistent TPT-use resulting in a cut-off ≥60% (χ^2^(4) = 61.26, *P* < 0.001), with reasonable AUC 0.78. There was a significant association for TPT use (yes/no) and risk behavior (low vs. high) defined from the algorithm (χ^2^(1)=58.6, *P* < 0.001). The algorithm had a high sensitivity, classifying 89% of non-TPT users correctly, while having a low specificity, classifying 49% of TPT-users correctly. This was confirmed by cross-validation (*n* = 34), reporting a high sensitivity (83%), despite a lower AUC (0.61).

**Discussion:**

The algorithm classifies high-risk inconsistent TPT-users with reasonable accuracy, but lacks the specificity to classify consistent users at low risk. This approach should be useful in identifying athletes that would benefit from additional counseling.

## 1 Introduction

Contaminants have been found in 19% of nutritional supplements sold in the United States (1), and recently, 38% of tested supplements contained undeclared doping substances in the Netherlands (2). In the United States, the number of doping violations with sanctions associated with supplements ranges from 0 to 17% of total investigated cases per year and has averaged around 9% of all doping cases annually since 2006 (3). One of the risk mitigation strategies to ensure athletes use safe nutritional supplements free from doping-related substances is to use products that are third-party tested (TPT) (4, 5). Despite the literature consistently mentioning the need for the use of TPT supplements (6, 7), research shows that a substantial number of athletes report inconsistently using TPT supplements (8, 9).

A reason for this low TPT compliance may be that current certification programs only cover 4–12% of the nutritional supplements currently on the market (10, 11); in addition, many of these programs do not test for banned substances. The large number of certification programs holding different standards makes it difficult for athletes to select programs that test for WADA-prohibited substances (12, 13). In addition, sports nutrition and dietary supplement knowledge are low in athletes (8, 14, 15), and access to nutrition experts, such as a sports RD, may be limited (8, 16). Overall, the endorsement of TPT nutritional supplements does not necessarily result in athlete compliance: despite many athletic departments instituting strict liability for drug testing, only 50–80% of athletes report the regular use of TPT supplements (8, 9).

Even though instituting strict liability in sport for drug testing (6, 7, 17), data suggest that 20–50% of athletes are not using TPT nutritional supplements, with high level (inter)national athletes reporting a 50% compliance with purchasing TPT supplements, vs. 67% in collegiate athletes and 80% in Olympic athletes (8, 9). These figures fall short of the 100% compliance goal of sports organizations. As to athlete attitudes, athletes normally consider it unacceptable to consume banned substances through supplements without their knowledge (9). Further, almost all student-athletes (93% based on *n* = 138) at a DI NCAA athletic department stated that it is important to know if supplements are tested for banned substances (8), while at the same time, 43% reported not using certified supplements (8).

Preventing the consumption of adulterated products containing substances not listed on the label is essential to limit exposing users to doping-related substances and health risks, as contaminated supplements may contain substances that negatively affect health (2). Current nutrition education curricula within athletics may improve the quality of dietary intake, but they contain little information about supplements and TPT programs (18, 19), and it is mainly the TPT programs that have their own quality assurance program testing for substances prohibited in sport that athletes should seek out (5). At the same time, the TPT programs need to be ISO 17025 accredited ensuring that the lab is deemed to be technically competent to adhere to the needed standards for lab testing, and participating brands need to adhere to their certification criteria (5). While previous research has assessed athletes’ knowledge (14, 15) and use of supplements (20–23), predictors for safe supplement use through the use of third-party tested supplements are currently unknown.

Therefore, the objective of this study was to develop an algorithm to predict whether athletes use TPT supplements while using a questionnaire assessing self-reported nutritional supplement behavior.

## 2 Materials and methods

### 2.1 Study design

This cross-sectional cohort design study asked athletes from six NCAA Division I athletic departments, from October 2022 to April 2023, to fill out a questionnaire about behavior and attitudes concerning safe nutritional supplement use, including a predefined nutritional supplement list to identify supplement and third-party tested (TPT) nutritional supplement use during the last 12 months. Athletes were recruited by email, at fuel stations, or during team meetings. The results were analyzed aiming to identify predictors for TPT supplement use that could be combined in one algorithm predicting the use of uncertified nutritional supplements vs. TPT nutritional supplements, resulting in a “supplement risk behavior score.” After identifying variables predicting TPT supplement use while using the full dataset, responses from participants not delivering all answers to each of the identified predictors were removed. Then, the remaining responses were randomly assigned to two datasets, a “training dataset” and a smaller “validation dataset” to develop and confirm the validity of the algorithm predicting TPT nutritional supplement use. The algorithm was created while analyzing the outcome of the training dataset using the weight (i.e., “Estimate”) and direction of the “estimate” outcome of the logistic regression analyses. See the Supplementary material for the original questionnaire (Supplementary File 1, Original Nutritional Supplement Survey), the supplement safety screener containing the algorithm-based questions (Supplementary File 2A, S3 Nutritional Supplement Screener or Supplementary File 2B, Qualtrics file), and an Excel file with instructions for calculation of the supplement risk behavior score and interpretation of the S3 screener (Supplementary File 3, S3 Instruction, work file, and interpretation).

### 2.2 Study participants

The participating NCAA Division I athletic departments, representing the highest-level collegiate athletes within the United States, included an estimated total of 3,580 student-athletes, ranging from 480 to 900 student-athletes per athletic department. Respondents had to be at least 18 years of age but under the age of 35, and a current member of a varsity sport at one of the participating athletic departments, while responding to at least 70% of the questionnaire. In cases of duplicate responses, the first response (identified via timestamp) was kept. Bot-generated responses were excluded before the initial response rate of 14% (*n* = 506) was calculated.

The study was approved by the Arizona State University Institutional Review Board (STUDY00015034). Student-athletes read and checked informed consent before accessing the questionnaire. The questionnaire, accessible through a link or QR-code, was anonymous, and upon completion, student-athletes were linked to a separate questionnaire where personal information would be provided and where they received a $17.50 virtual gift card for completion of the questionnaire.

### 2.3 Questionnaire

The web-based questionnaire, was administered through Qualtrics (SAP, Seattle, WA, USA) for which each question required a response to move forward. The questionnaire, with 85 questions, was partly adapted from published literature (8, 9, 24–26), with additional newly formulated “original” questions.

The questionnaire (Supplementary File 1), consists of five main categories:

General questions: Athletic department [#1], primary sport [#1], sex [#1], age [#1], athlete status [#1]—subtotal: 5 questions.

Information sources: Nutrition information and counseling [#1], contact moments [#1], topics addressed [#1], preferred health professional [#1], preferred information source [#1], types of social media use [#1], social media frequency [#1], daily time spent on social media per day [#1], social media use related to nutritional supplements and sports foods [#1], preferred way of contacting in case of new information [#1]– subtotal: 10 questions.

Supplement knowledge: Supplement section of the nutrition for sport knowledge questionnaire (NSKQ) [#12], supplements related to doping [#1], WADA familiarity [#1], contamination [#1], implications of failed drug test [#1]– subtotal: 16 questions.

Nutritional supplement use: Age of first use [#1], purchase outside athletic department [#1], frequency of TPT supplements during last 12 months [#1], who purchases supplements [#1], location of supplement purchase [#1], estimated contamination of supplements [#1], predefined supplement checklist [#1], TPT of individual supplements [#1], TPT logo recognition [#1]– subtotal: 9 questions.

Attitudes and barriers: Find and order TPT supplements [#1], common feelings and beliefs about TPT supplements [#11], strategies for safe supplement use [#1], solutions and purchases of (safe) supplement use [#18], personality traits [#14]—subtotal: 45 questions.

For this article, only the results of the questions that directly related to self-reported personality traits concerning the use or non-use of certified TPT nutritional supplements are reported.

### 2.4 Sample size

The NCAA reports that there are 460,000 NCAA DI-III student-athletes (27). For this purpose, a confidence level of 95% was used with a margin of error of 5%, while estimating that at least 50% of athletes use a third-party testing system while purchasing nutritional supplements (9). This resulted in a minimum number of 384 participants needed. This method of using margin of error was applied since the primary objective was to survey a sample of participants and have a large enough sample size to represent the intended population. This method of margin of effort prioritizes representativeness of the sample to the population over obtaining a sample size for a statistical analysis to detect a specific effect size. The latter is characteristic of research testing a specific hypothesis.

### 2.5 Statistical analysis

The demographics and descriptive data for relevant questionnaire sections are reported as percentages (%) and frequencies (*n*). For the development of the predictive model, cross-validation was performed using a 90:10 training/validation split. The purpose of adopting the 90:10 sample split for the cross validation was to target as large of a sample for the training dataset in order to establish a viable predictive model, while simultaneously securing a minimum of 30 complete responses in the validation sample so that the central limit theorem could be applied to a sample that was likely to be undersized in this initial stage of predictive modeling. This resulted in 320 participants in the training data without missing values. A stepwise logistic regression was run on each of the survey questions in the training dataset, while controlling for respondent sex, to determine which questions were related to the outcome of consistent vs. inconsistent TPT supplement use. For this analysis, *p* ≤ 0.10 was the initial threshold for retaining variables in the model. A final logistic regression model was run on the cross-validation dataset in which variables *p* < 0.05 were retained, with causal priority being granted to variables deemed practically relevant from a sports nutrition perspective in relation to selecting (safer) nutritional supplements. Model parameter estimates were obtained by including multiple predictor variables (as combined in the final algorithm) to evaluate the unique variance contribution of each survey question. These metrics were evaluated to ensure that each survey question contributed unique variance and predictive value above and beyond all other survey questions in the model. This process resulted in the final model, as presented in Table 1.

**TABLE 1:** Demographics of the NCAA Division I collegiate athletes surveyed reported as frequency (% and *n*) or as mean ± standard deviation, for total as well as training and cross-validation groups.

	Total group (*n* = 410)	Training data (*n* = 320)	Validation data (*n* = 34)
**Sex**			
Female	53% (*n* = 217)	52% (*n* = 166)	59% (*n* = 20)
Male	47% (*n* = 193)	48% (*n* = 154)	41% (*n* = 14)
**Age**			
Years	21.4 ± 1.6	21.5 ± 1.6	21.4 ± 1.71
**Athlete type (student-athletes could select multiple options)^‡^**			
Student-athlete at a collegiate AD	97% (*n* = 397)	98% (*n* = 314)	94% (*n* = 32)
Member of national team or selection	8% (*n* = 32)	8% (*n* = 25)	18% (*n* = 6)
Part of national doping testing pool	3% (*n* = 14)	3% (*n* = 8)	12% (*n* = 4)
Carded athlete	2% (*n* = 8)	2% (*n* = 6)	–
Student-athlete not at a US collegiate AD	1% (*n* = 5)	1% (*n* = 3)	–
Professional athlete	1% (*n* = 4)	1% (*n* = 2)	6% (*n* = 2)
Other	1% (*n* = 4)	1% (*n* = 2)	6% (*n* = 2)
**Have received nutrition information, counseling, or advice during the last 12 months^‡^**			
Sports RD within AD	89% (*n* = 366)	92% (*n* = 294)	94% (*n* = 32)
Sports RD outside of AD	8% (*n* = 33)	8% (*n* = 24)	12% (*n* = 4)
Other	2% (*n* = 9)	2% (*n* = 7)	–
I have not received nutrition information	8% (*n* = 31)	6% (*n* = 18)	6% (*n* = 2)

^‡^Percentages may not add to 100% as student-athletes could select multiple options. A total of *n* = 56 respondents were not reporting the use of nutritional supplements (*n* = 36) or did not provide all information need to be included in the analyses (*n* = 20).

Then the risk behavior score quadrants (i.e., 0–19%, 20–39%, 40–59%, 60–79%, 80–100%) were plotted against consistent vs. inconsistent TPT use to assess potential cut-off values in determining low vs. high risk to determine the best fit for the model, and chi-square analysis was used to determine significance. Best fit in this case means that it should best predict consistent vs. inconsistent third-party tested supplement use compared with other predictor variables. Finally, after establishing the algorithm predicting low vs. high risk of TPT, a chi-square test of association was run to estimate the relationship between the use of TPT products and the predicted binary risk grouping of using TPT products. The creation of a binary risk grouping of high vs. low risk was intended to show a simplified approach of categorizing risk. Finally, the fit of the model was assessed by calculating the area under the curve (AUC), as well as true positive (TP), false positive (FP), false negative (FN), true negative (TN) scores. These values were used to calculate sensitivity (TP/TP+FN) and specificity (TN/TN+FP), as well as positive predictive value (PPV = TP/TP+FP) and negative predictive value (NPV = TN/TN+FN).

The training model was tested on the validation data to confirm its replicability and reduce the likelihood of an overfit final model. Significance was set for *P* < 0.05 if not described differently.

## 3 Results

### 3.1 Demographics and response

**FIGURE 1:**
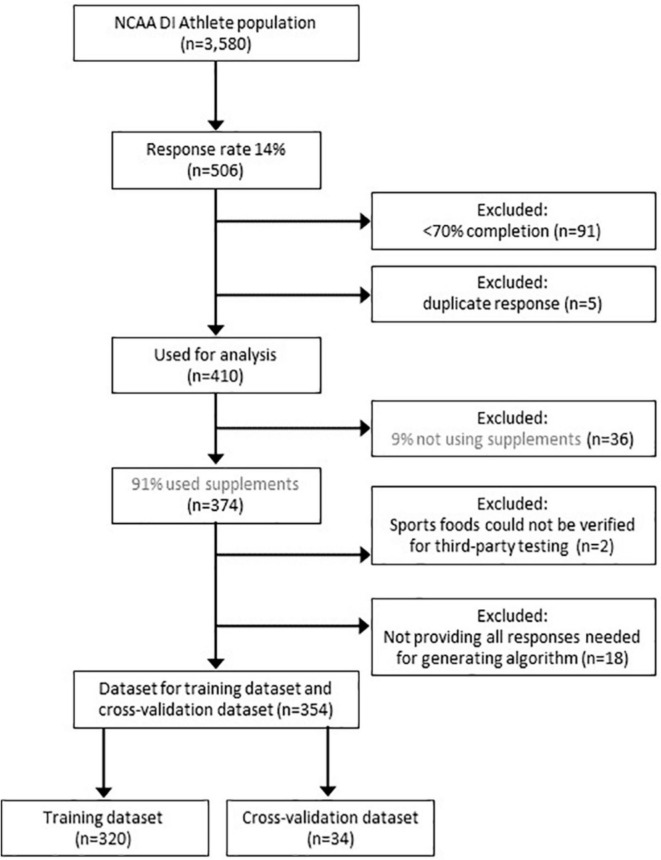
Consort diagram. The figure shows the response rate of NCAA Division I student-athletes, the reported use of nutritional supplements, as well as the number of responses that were excluded before constructing a training dataset and cross-validation dataset.

As shown in Figure 1, out of *n* = 506 responses a total of 91 questionnaires were excluded because athletes completed < 70% of the questions, and 5 additional questionnaires were excluded because of duplicate responses. Table 1 reports the demographics of the *n* = 410 NCAA Division I student-athletes (of which 53% were female, age 21.0 years or older, and IQR: 20.0 to 22.3, from > 20 sports) including those not reporting the use of nutritional supplements (9%, *n* = 36), as well as student-athletes reporting at least one, but often more, nutritional supplement (91%, *n* = 374). In addition, Table 1 shows also the demographics for the training dataset (*n* = 320) and cross-validation dataset (*n* = 34) that were created after removal of participants reporting sports foods of which the need for TPT could not be verified (< 1%, *n* = 2), and after removing a small number (4%, *n* = 18) of participants that did not provide all responses for the questions needed for the risk behavior algorithm.

### 3.2 Development of the algorithm using the training dataset

#### 3.2.1 Training dataset description

Based on a 90:10 training validation split, the data of a total of *n* = 320 participants were randomly selected for the training dataset for the development of the algorithm and to determine the best fit to classify a high vs. a low risk of not using TPT supplements. A total of 38% (*n* = 121) consistently used TPT vs. 62% (*n* = 199) inconsistently using TPT.

#### 3.2.2 TPT supplement use predictors

Table 2 contains 10 items that were significantly associated with TPT risk (i.e., consistent vs. inconsistent TPT-use) with *P* ≤ 0.10, it was already mentioned that these items were based on the 85 questions and the two newly created variables. Out of these 10 items, four items were part of the predefined supplement question list (i.e., multivitamin, weight gainer, caffeine, and creatine). Despite that the Wald chi-square outcome for multivitamin use was *P* = 0.1116, the supplement was included as it is one of the most popular dietary supplements (46%, *n* = 190), together with caffeine (56%, *n* = 229), creatine (22%, *n* = 91), and weight gainer (5%, *n* = 22).

**TABLE 2:** Variables identified as predictors whereas athletes are using TPT nutritional supplements, including the prevalence per outcome for *n* = 320 NCAA Division I collegiate athletes.

Variable	Prevalence (% yes)	DF	Estimate	Standard error	Wald chi-square	Pr > chi-square
Intercept	–	1	0.54	0.36	2.23	0.136
WADA familiarity: Are you familiar with banned substances that may occur in nutritional supplements listed on the WADA (world anti-doping agency) list? (select only one—yes/no)	52%	1	−0.49	0.28	3.02	0.082
Knowing where to find & order TPT supplements: I know where to find and order third-party tested supplements (only select one—yes/no).	50%	1	−0.64	0.30	4.57	**0.033**
Search for information: Where do you go to look for information on nutritional supplements and sports foods? (check all that apply—checked score: I do not search for information on my own)	22%	1	0.81	0.36	5.15	**0.023**
Discussing supplement choices with RD: I discuss all my supplement choices with the Athletic Departmental Sports RD (only select one—yes/no).	58%	1	−0.81	0.28	8.24	**0.004**
Purchase outside athletic department: Do you purchase or use nutritional supplements outside what is provided by your Athletic Department? (only select one—yes/no)	49%	1	0.53	0.29	3.33	**0.068**
Advise from others: I’ve decided to purchase one or more supplements as a result of the advice of family, friends, or teammates (only select one—yes/no).	53%	1	0.57	0.28	4.19	**0.041**
**Please check all of the following nutritional supplements you have used during the last 12 months (check all that apply).**						
Multivitamin: (multivitamin and mineral supplement checked)	51%	1	−0.44	0.27	2.53	0.112
Weight gainer: (weight gainer checked)	6%	1	−0.96	0.56	2.89	**0.089**
Caffeine: (caffeine checked)	62%	1	1.34	0.28	22.89	**< 0.0001**
Creatine: (creatine checked)	25%	1	−0.62	0.33	3.55	**0.059**

Logistic regression was used to identify predictors and Wald chi-square was calculated to indicate significance, with *P* ≤ 0.10 (indicated in bold font). The multivitamin variable was borderline significant, but because of its prevalence, the supplement was still discriminatory and adding in a meaningful way to the algorithm. The variables listed in the table delivered the following algorithm predicting TPT supplement use: 2.72 ^ ( (0.54) + (−0.48*WADA) + (−0.64*FIND&ORDER) + (0.81*SEARCH) + (−0.81*DISCUSSWITHRD) + (0.53*PURCHASEOUTSIDE) + (0.57*FRIENDADVICE) + (−0.43*MULTIVITAMIN) + (−0.96*WEIGHTGAINER) + (1.34*CAFFEINE) + (−0.62*CREATINE)) /1 + 2.72 ^ ( (0.54) + (−0.48*WADA) + (−0.64*FIND&ORDER) + (0.81*SEARCH) + (−0.81*DISCUSSWITHRD) + (0.53*PURCHASEOUTSIDE) + (0.57*FRIENDADVICE) + (−0.43*MULTIVITAMIN) + (−0.96*WEIGHTGAINER) + (1.34*CAFFEINE) + (−0.62*CREATINE)).

#### 3.2.3 Risk behavior score

The algorithm supporting the risk behavior score was based on the weight (i.e., “Estimate”) and direction of the “estimate” outcome as mentioned in Table 2. The variables listed in the table delivered the following algorithm predicting TPT supplement use:

$$\frac{2.72^{\begin{array}{cc} (\left(0.54\right)+\left(-0.48*W\right)+\left(0.64*FO\right)+\left(0.81*S\right)+ & \\ \left(-0.81*D\right)+\left(0.53*P\right)+\left(0.57*FA\right)+(-0.43 & \\ {}*M)+\left(0.96*W\right)+\left(1.34*CA\right)+\left(-0.62*CR\right)) & \end{array}}}{1+2.72^{\begin{array}{cc} (\left(0.54\right)+\left(-0.48*W\right)+\left(-0.64*FO\right)+\left(0.81*S\right) & \\ +(-0.81*D+\left(0.53*P\right)+\left(0.57*FA\right)+(-0.43 & \\ {}*M)+\left(-0.96*W\right)+\left(1.34*CA\right)+\left(0.62*CR\right)) & \end{array}}}$$

In which capitalized letters reflect the following question items:

W = WADA; FO = FIND & ORDER; S = SEARCH; D = DISCUSS WITH RD; P = PURCHASE OUTSIDE; FA = FRIEND ADVICE; M = MULTIVITAMIN; W = WEIGHTGAINER; CA = CAFFEINE; CR = CREATINE.

This algorithm suggests an increased risk of not using TPT, with a higher score ranging from 0 to 1, in which a low score suggests a low risk vs. a high score suggesting a high risk.

#### 3.2.4 Determining the best cut-off for low vs. high risk of not using TPT

To find the best fit indicating a low vs. high risk for inconsistent or not using TPT, risk behavior score quadrants were plotted against consistent vs. inconsistent TPT use to determine the best-fit cut-off value to determine low vs. high risk, as shown in Table 3. This table shows that the discriminatory cut-off was between quadrants three and four at the 60% mark.

**TABLE 3:** Algorithm-based risk behavior score for using vs. not using TPT quadrant against consistent vs. inconsistent TPT-use analysis for *n* = 320 NCAA Division I collegiate athletes.

TPT-use	Risk behavior score quadrants: 1–5 (with % indicating algorithm outcome)					
	1 (0–19%)	2 (20–39%)	3 (40–59%)	4 (60–79%)	5 (80–100%)	Total
Consistent (%, *n*)	2%	3%	14%	18%	1%	38%
	6	10	43	58	4	121
Inconsistent (%, *n*)	0%	1%	5%	50%	6%	62%
	0	4	17	159	19	199
Total (%, *n*)	2%	4%	19%	68%	7%	100%
	6	14	60	217	23	320

Chi-square analysis for risk quadrant analysis for consistent vs. inconsistent TPT-use was significantly different [χ^2^(4) = 61.26, *P* < 0.001] suggesting a good discriminatory ability for the risk behavior score.

#### 3.2.5 Categorized results for low vs. high risk of not using TPT

When classifying athletes for consistent/inconsistent TPT use according to questionnaire-based self-reporting and low/high-risk behavior based on the algorithm, only the athletes reporting all predictive variables could be included in the algorithm. When applying the results of the *n* = 320 student-athletes that provided answers for the variables based on the final model for the algorithm, a total of 38% (*n* = 121) consistently reported the use of TPT supplements, meaning that all the supplements they used were TPT. The rest of the athletes inconsistently used TPT (62%, *n* = 199).

As shown in Table 4 the screener was able to identify significantly different responses [χ^2^ (1) = 58.59, *P* < 0.001], indicating a strong relationship between the risk groupings and product use outcomes. The screener had a high sensitivity, the test’s ability to designate an individual with a negative outcome, as it classified 89% of the student-athletes not consistently using TPT accurately in the ≥ 60% high-risk behavior score group. Despite the test’s high sensitivity, the test had a low specificity (49%), as roughly half of the consistent TPT users were correctly classified in the < 60% low-risk behavior score group. The area under the curve (AUC) for the cut-off value ≥ 60% suggesting a high risk for inconsistent TPT was 0.78, with a positive predictive value (PVV) of 0.69, and negative predictive value (NPV) of 0.71.

**TABLE 4:** Training data absolute and relative categorized screener-based answers for consistent/inconsistent TPT use vs. algorithm-based low/high-risk behavior toward TPT supplement use in *n* = 320 NCAA Division I collegiate athletes.

	< 60% algorithm score “Low risk”	≥ 60% algorithm score “High risk”	Total
Consistent TPT-use, % (*n*)	18% (59)	19% (62)	38% (121)
Inconsistent TPT-use, % (*n*)	7% (21)	56% (178)	62% (199)
(sub)Total, % (*n*)	24% (83)	76% (257)	100% (320)
Consistent TPT-use (Positive, *n*)	TN	FP	Specificity
	59	62	49%
Inconsistent TPT-use (Negative, *n*)	FN	TP	Sensitivity
	21	178	89%

Chi-square analysis for consistent/inconsistent TPT-use vs. algorithm-based low/high-risk behavior toward TPT supplement use was significantly different [χ^2^(1) = 58.59, *P* < 0.001] suggesting a good discriminatory ability for the selected ≥ 60% risk behavior score cut-off. FP, false positive; TN, true negative; FN, false negative; TP, true positive.

### 3.3 Cross-validation outcomes

#### 3.2.1 Cross-validation dataset description

Based on a 90:10 training validation split, the remainder of *n* = 34 participants were used to validate the model from the training data in a cross-validation. A total of 29% (*n* = 10) consistently used TPT vs. 71% (*n* = 24) inconsistently using TPT.

#### 3.2.2 Categorized results using cross-validation data for low vs. high risk of not using TPT supplements

When applying the same strategy as for the training data for the cross-validation data (Table 5), the crosstab table had very similar percentages to the training data shown in Table 4; however, the chi-square analysis was not significant [χ^2^(1) = 2.14, *P* = 0.144]. This non-significant relationship indicates that the binary risk categories did not significantly predict product use. This lack of relationship, however, could be a result of the small size of this sample. Still, the screener had a similar high sensitivity as the training dataset, as it classified 83% of the student-athletes inconsistently using TPT accurately in the ≥ 60% high-risk behavior score group. In addition, the validation data also reported a low specificity while including only 40% of the consistent TPT users in the < 60% low-risk behavior score group identifying them as “low risk.” The AUC for the cut-off value ≥ 60%, suggesting a high risk for inconsistent TPT, was lower than in the training data with 0.61, and a PVV of 0.77 and NPV of 0.50.

**TABLE 5:** Cross-validation absolute and relative categorized screener-based answers for consistent/inconsistent TPT use vs. algorithm-based low/high-risk behavior toward TPT supplement use in *n* = 34 NCAA Division I collegiate athletes.

	< 60% algorithm score “Low risk”	≥ 60% algorithm score “High risk”	Total
Consistent TPT-use, % (*n*)	12% (4)	18% (6)	29% (10)
Inconsistent TPT-use, % (*n*)	12% (4)	58% (20)	71% (24)
(sub)Total, % (*n*)	24% (8)	76% (26)	100% (34)
Consistent TPT-use (positive, *n*)	TN	FP	Specificity
	4	6	40%
Inconsistent TPT-use (negative, *n*)	FN	TP	Sensitivity
	4	20	83%

Chi-square analysis for consistent/inconsistent TPT-use vs. algorithm-based low/high-risk behavior toward TPT supplement use was significantly different [χ^2^(1) = 2.14, *P* = 0.144] suggesting a good discriminatory ability for the selected ≥ 60% risk behavior score cut-off. FP, false positive; TN, true negative; FN, false negative; TP, true positive.

## 4 Discussion

### 4.1 Recap of the study outcomes

This algorithm was determined to be very suitable for identifying inconsistent TPT users indicated by a ≥ 60% high-risk behavior score. For those consistently using TPT supplements, the algorithm provides more insight into their behavior, but due to its low specificity it cannot replace the actual checking of self-reported TPT use to identify consistent TPT users.

### 4.2 Predictor variables

The developed algorithm was based on ten predictive variables including four nutritional supplements that were clearly related to TPT use. Six elements (WADA familiarity, knowing where to find and order TPT supplements, discussing supplement choices with a sports RD, and using multivitamins, weight gainer, and creatine) were associated with consistent TPT use, while the other four elements (not searching for information, purchasing products outside of the supplements provided by the athletic department, deciding to purchase supplements based on the advice of others, such as family, friends or team mates, and using caffeine) were associated with inconsistent TPT use. This section discusses, where possible, the relevance of these predictors.

To start, the majority of athletes participating in high level competitive sports need to comply with WADA regulations (28). Not complying may result in a positive doping test (4). Supplements often contain impurifications, that can be on the WADA (or in this case NCAA) prohibited substances list. This can lead to a positive doping test, hence the development of third-party testing organizations that batch test supplements (5). The analyses in the current study showed that there was indeed an association between athletes being familiar with the prohibited list from WADA and consistent TPT use. Potentially this can be related to access to a sports dietitian or sport level, or both. Despite roughly half of the athletes in the current study reporting familiarity with the WADA prohibited substance list, others may be less familiar with it. This includes for example semi-professional football players in South Africa, of which almost all (87%), never attended a workshop on safe supplement use, while reporting WADA familiarity ranging from 16% (29). On the contrary, up to 95% in Japanese Olympic and Paralympic athletes reported familiarity with the WADA prohibited substance list, still half of them used supplements without the advice of a doctor or dietitian (30).

Subsequently, being able to find and order TPT supplements was also associated with consistent TPT use, as it has been previous reported that only 22% of high school athletes knew where to find TPT supplements and only 25% of these athletes knew where to order TPT supplements, resulting in 24% reporting consistent TPT use (31). In a follow-up study on a subsample of high school athletes at the same high school, it was shown that education indeed resulted in an increased intention to select TPT supplements (32).

Almost two-thirds of the athletes discussed supplement choices with their registered dietitian, which was the fourth predictive variable, being associated with a lower risk of not using TPT supplements. This can be related with better informed choices by athletes when counseled by a dietitian (16), while in reality athletes also report lower percentages for consulting a dietitian related to supplement use, ranging in other recent publications from 25% (33) to 50% (30).

Finally, the use of three nutritional supplements: multivitamin and mineral supplements, weight gainer, and creatine were associated with consistent TPT use. Interestingly, based on previous reporting multivitamin and mineral supplements (≥ 60%), and creatine (20–30%) are among the more popular supplements, whereas weight gainer supplements have been less popular (< 10%) (8, 16, 31). Earlier no relationship was seen for multivitamin and mineral use, and creatine use and having access to a sports dietitian (16), but at the same time all three supplements can be reasonably classified as potentially effective in optimizing nutrient intake, or in providing extra protein or creatine as supported by the current sports nutrition consensus (6). More research is needed to better understand why specifically these supplements were more clearly related to TPT use than others.

The remainder of the predictors (i.e., not searching for information, purchasing outside of the athletic department, taking advice from family and friends, and the use of caffeine supplements) suggest a negative impact on TPT use. The strongest impact of a variable influencing the algorithm came from the use of caffeine. There is not a clear literature-based reason supporting the relation between using caffeine and not consistently using TPT supplements, on the other hand caffeine was one of the most frequently reported supplements (62%), and as such, because it is so frequently used it may be associated with the larger part of the athletes not being consistent TPT users. In addition, caffeine can be found for example in pre-workout supplements which seem to be more prone to adulteration (34). The next strongest impact on the algorithm was based on athletes not searching for information before using a supplement. In the current study, one-fifth of the athletes reported not to search for information on their own, which was substantially lower than earlier reported, as 50% of Japanese elite Olympic athletes reported not reviewing scientific evidence before using nutritional supplements (30). Two other predictors, both contributing with a similar weight to the algorithm, where purchasing supplements outside of what the athletic department provides, as well as taking advice from non-experts, increased the chance of inconsistent TPT use. Regarding the element of purchasing supplements outside of what is provided by the department, it is important to acknowledge that supplements provided by the athletic department is something that specifically applies to NCAA DI collegiate athletics, and potentially elite athletics (35), because these athletic programs have a larger budget than within other divisions (36). The supplements provided within the departments are likely at close to zero risk because they have registered sports dietitians on staff that organize the products available. At the same time, purchasing products outside of the athletic department by the athlete itself is therefore always subject to risk. Due to the large offering of supplements, and the relatively small number of third-party tested supplements testing for substances prohibited in sport, local stores may not the best resource for third-party tested supplements. Athletes should therefore be trained to find and order TPT supplement from third-party testing organizations. Lastly, purchasing supplements based on advice from others (family, friends, teammates) increased the risk of not using TPT supplements. Unfortunately, based on the available literature, this is a very common influence (36–38). In general, friends and family as well as teammates can normally not be considered experts, as a result the advice to a supplement is unlikely to be evidence based, and it is not unlikely that the friends and family are not familiar with third-party testing supplement procedures. Therefore, it comes back to making sure athletes consult with an expert (16, 39), which is reflected in the algorithm as discussing supplement choices with RD was associated with TPT use, whereas deciding to purchase supplements based on the advice of others, such as family, friends or team mates was associated with inconsistent or no TPT use.

### 4.3 Criterion validity of the algorithm compared to self-reported TPT use

The current algorithm had a high sensitivity to detect inconsistent self-reported TPT use or no TPT use, while the algorithm had a low specificity because it marked roughly half of the consistent TPT users with a high-risk profile. Sensitivity and specificity are inversely related, and therefore when sensitivity increases, specificity tends to decrease, and vice versa (40). Therefore, it is important to identify what the main objective is of a tool. In this case, as the majority of athletes reports inconsistent TPT use, it’s more important to identify correctly the non-TPT users than it is to correctly identify the users of TPT supplements. This is, because the absolute numbers of correctly classified inconsistent TPT users will be higher. It also means that when the algorithm is used on its own, without asking the complete number of supplements used, it will result in a misclassification of roughly half of the consistent TPT users. Therefore, it is recommended to have a face-to-face follow-up with a specialized sports health professional (39), such as a sports dietitian or nutrition expert (16). Further, the sensitivity and specificity of this algorithm should be confirmed in future research in an independent dataset, while also looking for optimization of the algorithm to ensure a better accuracy of risk classification of athletes.

### 4.4 Athlete compliance to third-party testing and the need for screening

There is currently almost no data that reports the relation between TPT supplement use and knowledge, attitude and practices of athletes. The existing data shows that there is no clear relationship between knowledge and TPT supplement use (8, 31), but this may be related to generalized low knowledge scores of these athletes. Further, most athletes find it unacceptable that supplements can contain unlabeled substances that could lead to a positive doping test (9), but despite reflecting these attitudes a large group of athletes does not report to use third-party tested supplements (8, 9, 31). Hence, the relevance for developing a screener scoring risk behavior toward the consistent use or non-use of TPT nutritional supplements lies in that many athletes currently are not selecting third-party batch tested supplements. Although nutritional supplement education may play an important role (32), this study shows that even within athletic departments that provide access to sports dietitians and nutrition education, as well as providing nutritional supplements allowed per NCAA regulations, consistent TPT use is low (38%). At the same time TPT supplement use can be even lower, but likely TPT supplement use can also increase with the right education. While using a similar approach, using a predetermined list of nutritional supplements and asking per supplement if it was TPT, only 24% of high school athletes (14–19 years, *n* = 225) claimed to know for sure that all their supplements were third-party tested, with TPT nutritional supplement use ranging from 0 to 100% for individual supplements (31). A subset of this high school athlete population (*n* = 106) was later part of a study investigating the impact of a high school athlete education program for safe nutritional supplement use, and 35–77% of supplements reported were TPT, which increased after the education module 7–36% depending on individual supplements reported (32).

It is important to emphasize that earlier studies, of which two are listed below, have asked only a general question about third-party supplement use, not specifically related to individual supplement use. Self-reported compliance numbers for TPT supplement use in these cases are slightly higher, but may substantially overestimate TPT supplement compliance. For example, the United States Anti-Doping Association (USADA) published the results of their 2022 Athlete Perceptions Survey, covering *n* = 994 athletes from 76 sports (80% Olympic, 20% Paralympic), while asking a generic question about the use of NSF certified for sport supplement. Finding that 56% of the athletes reported always checking for NSF certified for sport certification (41), and although this was lower than the 82% self-reported compliance to TPT in Dutch athletes using the NZVT system (i.e., the Dutch Safeguards System for Dietary Supplements) (9), both cases likely overestimated consistent TPT use.

### 4.5 Strengths and limitations

A clear strength of this study is that the use of TPT supplements was questioned for individual supplements. Another strength was the large range of questions that was analyzed for its predictive value for TPT supplement use, while including a sample size representative for the NCAA DI student-athlete population. The response rate was with 14% higher than was anticipated, and after cleaning, the dataset reflected 11.5% of the sampled population, which was likely enough to accurately reflect the behavior of this student-athlete population, with an almost equal sex distribution, coming from six different athletic institutions throughout the United States, and develop the algorithm predicting TPT use.

At the same time, the study also holds limitations. The responses used for both the training dataset and cross-validation were minimal, and should be seen as a first step in confirming the predictive modeling process. The resultant small sample in the validation dataset may have yielded higher variability that could have a disproportionate effect, compared with actual model predictability, on reducing the model fit metrics of the validation data, such as AUC (42). As funds were a limiting factor, the cross-validation should be interpreted as a first pilot, therefore, it can be suggested that independent data collection in a much larger group of athletes should confirm these results in the future. Furthermore, it is unclear how well the algorithm can be translated to other (athlete) populations in less well funded athletic departments; for example, the ones that do not have access to supplements provided by an athletic department. At the same time, these athletes will need to select supplements on their own; consequently, they risk purchasing uncertified or not third-party tested supplements. Another limitation, as this is a first reporting of the development of this algorithm, is that no further stratifications have been performed.

Real-world limitations of this screener include the simplified perspective of questioning athletes (as the algorithm requires a binary yes/no answer), as well as the predefined list of supplements that likely needs to be updated regularly when supplement options and offerings change. Further, despite the limited number of questions and simple formatting of the screener it is not clear what the test-retest reliability is of this type of questionnaire. Finally, despite a reasonable sensitivity (allowing to identify inconsistent TPT-users), the specificity to identify consistent TPT-users correctly is low; therefore, the algorithm cannot currently used independently of questioning self-reported TPT use (which is identified as part 3 of the current screener), or without follow up from a specialist, such as a sports dietitian.

## 5 Conclusion

The algorithm classifies high-risk inconsistent TPT users with reasonable accuracy, but lacks the specificity to classify consistent users at low risk. Still, the algorithm can help to identify most of the inconsistent TPT users, allowing sports health professionals, such as sports dietitians and nutritionists to identify the needs of athletes not consistently using third-party tested nutritional supplements, and help athletes to select TPT supplements, while improving athlete compliance to athletic program policies and reduce doping risk. Further research is needed to have the algorithm function independently from self-reported supplement and TPT behavior in athletes.

## 6 The practical application: supplement safety screener

### 6.1 Material overview

The algorithm as described in this article has been integrated in a first version of a supplement safety screener (S3, Supplementary File 2) consisting of three parts: (1) general information; (2) Algorithm-based supplement risk behavior score; and (3) supplement use and self-reported use of TPT supplements. Additionally, materials were developed that will help to calculate the risk behavior score and to interpret the screener outcome, all included as Supplementary material (as tab in Supplementary File 2). The following sections briefly describe the content of the screener, followed by some suggestions for interpretation of screener outcome.

### 6.2 Screener content

The general information in the current screener in part 1 is limited to the athlete’s name and team or sport. This allows the sports dietitian/nutritionist to identify the athlete for future follow-up, while also helping to organize the data per team sport, in case the screener is collected via a web-based questionnaire module. The supplement risk behavior score in part 2 is based on the variables listed in Table 1 using the algorithm as listed in the legend of this table. This algorithm can also be found in the following Excel file included as Supplementary material. There were two questions added to the screener that are not included in the algorithm, as they pose relevance for the nutritionist. This includes a question about the “Influence of teammates in trying a supplement” (to determine potential player dynamics related to supplement use) and the “Nutritional supplement purchase location” (allowing to further discuss the best outlets for supplement purchase with the athlete). Aside from the contributions of these predictors to a risk behavior score, it is important to emphasize that the individual outcomes also can provide important information about risk behavior in athletes. Finally, in part 3, supplement use and self-reported use of TPT supplements is questioned. This list is based on the pre-defined list of nutritional supplements used in this study, but it is suggested that this list should be kept up to date, to ensure that the most accurate assessment of self-reported supplement use and TPT use is performed.

### 6.3 Use and interpretation of the screener

The screener combines the outcome of the risk behavior scores and TPT-use, based on the outcome an athlete reporting the use of nutritional supplements will fit in one of the combinations listed in Table 6. This will help the sports dietitian/nutritionist to act, while considering the supplement and risk behavior of each individual athlete that filled out the screener. The table does not address the actions for athletes not currently reporting the use of nutritional supplements, but they should be also educated on the importance of the future use of TPT supplements in case athletes decide to start using nutritional supplements.

**TABLE 6:** Screener-based interpretation and suggested actions for a low- vs. high-risk behavior and TPT supplement use for those reporting the use of nutritional supplements.

TPT classification	Risk behavior	
	Low < 60%	High ≥ 60%
Consistent use of TPT	Good—athlete has a low-risk behavior score while consistently using TPT supplements Action: No direct action needed.	Fair—athlete has a high-risk behavior score while consistently using TPT supplements Action: Address the high-risk behavior and discuss if it may influence future choices related to TPT use
Inconsistent use of TPT	Fair—athlete has a low-risk behavior score while inconsistently using TPT supplements Action: The athlete has low-risk behavior, discuss TPT options for relevant supplements	Poor—athlete shows a high-risk behavior score, and the athlete inconsistently uses TPT supplements Action: The athlete has high-risk behavior, discuss behavior and TPT options
No use of TPT	Poor—athlete has a low-risk behavior score while using supplements, but not using TPT supplements Action: Explain the need for TPT supplement use for relevant supplements	Poor—in addition to reporting a high-risk behavior score while using supplements, but the athlete is not using TPT supplements Action: Explain the need for TPT supplement use, address high-risk behavior

It is suggested that athletes who fall into the fair or poor categories should have a follow-up by a knowledgeable sports nutrition expert for education regarding TPT and safety.

It is also an option to ask only the questions of part 2. This results in a reduced screener length while being able to calculate a risk score. Specifically, when the score is ≥ 60%, this warrants follow-up for sure, but as sensitivity and specificity of the algorithm is not perfect, follow up from a specialist, such as a sports dietitian should be considered.

## Data availability statement

The datasets presented in this article may be requested upon an informal inquiry addressed to the corresponding author, who will decide on a case-by-case basis if the request can be granted. Requests to access the datasets should be directed to Floris.wardenaar@asu.edu.

## Ethics statement

The studies involving humans were approved by the Arizona State University Institutional Review Board (STUDY00015034). The studies were conducted in accordance with the local legislation and institutional requirements. The participants provided their written informed consent to participate in this study.

## Author contributions

FW: Conceptualization, Data curation, Formal analysis, Funding acquisition, Investigation, Methodology, Project administration, Resources, Software, Supervision, Validation, Visualization, Writing – original draft, Writing – review & editing. KS: Data curation, Formal analysis, Investigation, Project administration, Writing – review & editing. RS: Conceptualization, Formal analysis, Methodology, Validation, Writing – review & editing. CG: Conceptualization, Funding acquisition, Methodology, Supervision, Writing – review & editing.
