# Supplementary material for: Development of a screener to assess athlete risk behavior of not using third-party tested nutritional supplements
Source: Front Nutr. 2024 May 15;11:1381731. doi: 10.3389/fnut.2024.1381731 (PMC11134207; doi:10.3389/fnut.2024.1381731)
Supplement: Supplementary file 1 [file Data_Sheet_1.zip › Supplemental File 1 Original Nutritional Supplement Survey.docx]

| Note. This is the word document version of the survey that was used in the published study with the title: “Development of a screener to assess athletes risk behavior toward not using third-party tested nutritional supplements increasing positive doping testing risk” by Wardenaar et al. 2024. Organizations working with Qualtrics can reach out to [Floris.wardenaar@asu.edu](mailto:Floris.wardenaar@asu.edu) for the QSF file of the survey. We are always open to discuss collaboration on future projects planning to use (elements of) this questionnaire. In addition, we ask organizations using the questionnaire to refer to the original published study. |
| --- |

Nutritional Supplement Survey - CPSDA

Start of Block: Introduction

Q1 **Introduction**

Q2
Dear Athlete, 
 
Thank you for being part of this study in which we investigate the knowledge, motivations and attitudes, and barriers associated with safe nutritional supplement use in a collegiate athletic population. It will take 15-20 minutes of your time, depending on the answers that you provide. 
 
After signing this informed consent section the questionnaire covers multiple topics like: general questions, nutritional supplement use, knowledge about supplements,  attitudes and barriers towards nutritional supplement use, as well as some personality traits. 
 
The following definitions are used about supplements in this questionnaire: 
 
**Nutritional supplements:** The combination of all dietary supplements, sports foods, and ergogenic supplements. 
 
**Dietary supplements:** Are considered vitamins, minerals, and essential fatty acids.


**Sport foods:** Are considered sports drinks, protein shakes, and sports bars. 
 
**Ergogenic supplements:** Are considered supplements that most of the time are not provided by your Athletic Department or Sport Organization (such as creatine) that go with a performance-enhancing claim. Keep in mind that an ergogenic supplement's definition is based on its claim and not necessarily on the evidence of its efficacy. 
 
To receive your incentive, you will need to provide a complete questionnaire, followed by your personal information (first and last name, and a valid university email address) that you can provide in a separate follow up questionnaire. As soon as we receive your full response we will start processing the incentive. Normally, you will receive your incentive within 7 work days after filling out the questionnaire. Make sure to claim the gift card ($17.50) after you receive it. 
 
Thank you for your time. 
 
Kind regards, on behalf of the research team,   
 


Dr. Floris Wardenaar, Principle Investigator, Arizona State University
Ms. Kinta Schott, Arizona State University

| Page Break |  |
| --- | --- |

Q3 **Signing informed consent**

Q4 INTRODUCTION
The purpose of this form is to provide you (as a prospective research study participant) information that may affect your decision as to whether or not to participate in this research and to record the consent of those who agree to be involved in the study. 
 
RESEARCHERS
 Dr. Floris Wardenaar an Assistant Professor in the College of Health Solutions at Arizona State University, as well as your athletic department have invited your participation in a research study that includes one questionnaire.

 STUDY PURPOSE
 Investigate the knowledge, motivations and attitudes, and barriers associated with safe nutritional supplement use in a collegiate athletic population, as well as describing current sport body-initiated education efforts and relate the outcomes to collegiate athlete nutritional supplement choices. 
 
DESCRIPTION OF RESEARCH STUDY
 You have received a link to this web-based questionnaire, you will be asked to fill out this informed consent each time, and after this fill out questions from multiple categories like general questions, nutritional supplement use, knowledge about supplements, attitudes and barriers towards nutritional supplement use, as well as some personality traits. 


RISKS
 We anticipate that there are no risks to the participants for filling out this questionnaire. 
 
BENEFITS
 The researchers cannot provide you with personal feedback, but the overall results will be shared with your sport organization which may benefit you in the future. 
 
NEW INFORMATION
 If the researchers find new information during the study that would reasonably change your decision about participating, then they will provide this information to you. 
 
CONFIDENTIALITY
 All information obtained in this study is strictly confidential unless disclosure is required by law. Dr. Wardenaar and his team will oversee data collection and storage at ASU using Qualtrics, a data collection tool and database. To ensure privacy and confidentiality, data will be secured during storage, use, and transmission by training all research team members working on this project and restricting authorization of access to the research team members. All electronic data will be stored on a secured, firewall and password-protected ASU server; no identifiable hard copy information will be created or stored. The results of this research study may be used in reports, presentations, and publications, but the researchers will not identify you. 
 
WITHDRAWAL PRIVILEGE
 It is OK for you to decide not to proceed with filling out this questionnaire. Even if you decide to start filling out the questionnaire, you may stop and withdraw from the study at any time. Your decision will not affect your relationship with Arizona State University or with the institution that asked you to fill out this questionnaire and this will not cause a loss of benefits to which you might otherwise be entitled.

 COSTS AND PAYMENTS
 The researchers want your decision about participating in the study to be absolutely voluntary. There are no costs for participating in this research. There is a $17.50 US dollar incentive for each completed questionnaire. This incentive will be sent to your email address within 7 work days after the questionnaire is marked complete by the research team. 
 
VOLUNTARY CONSENT
 Any questions you have concerning the research study or your participation in the study, before or after your consent, will be answered by Floris Wardenaar, floris.wardenaar@asu.edu or +1 (480) 599-5945. If you have questions about your rights as a subject/participant in this research, or if you feel you have been placed at risk; you can contact the Chair of the Human Subjects Institutional Review Board, through the ASU Office of Research Integrity and Assurance, at 480-965 6788. This form explains the nature, demands, benefits and any risk of the project. By signing this form, you agree knowingly to assume any risks involved. Remember, your participation is voluntary. You may choose not to participate or to withdraw your consent and discontinue participation at any time without penalty or loss of benefit. In signing this consent form, you are not waiving any legal claims, rights, or remedies. A copy of this consent form will be offered to you. 
 
Athletes need to be 18 years or older to be included in this study.

 

| 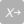 |
| --- |

Q5 To ensure your personal information is collected separately from the answers you provide you will be directed to another questionnaire at the end of this survey allowing you to fill out your first and last name, and email address.

Identify below if you consent to participate in this study:

- I consent to participate (please forward to the next part of the survey) (1)

| Page Break |  |
| --- | --- |

Q6 The questionnaire is broken up into small sections to increase readability. Make sure you read each question carefully before you insert your answer. You will not be able to correct answers after you have pushed the "next" button

End of Block: Introduction

Start of Block: General questions

Q7 **General questions**

| 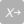 |
| --- |

Q8 What is your Athletic Department?

▼ Arizona State University (1) ... Louisiana State University (6)

| 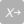 |
| --- |

Q9 What is your primary sport?

▼ Artistic Swimming (1) ... Dance (W) (44)

| Page Break |  |
| --- | --- |

| 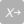 |
| --- |

Q10 What is your sex?

▼ Female (1) ... Intersex (3)

| 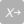 |
| --- |

Q11 What is your current age? (years)

▼ 17 (1) ... 45 (29)

| Page Break |  |
| --- | --- |

| 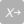 |
| --- |

Q12 Check all that apply concerning your athlete status. (Check all that apply)

- Carded athlete (1)
- Part of a national doping testing pool (2)
- Member of a national team or selection (3)
- Student-athlete at a US collegiate athletic department (4)
- Student-athlete not at a US collegiate athletic department (5)
- Professional athlete (6)
- Other (7)

End of Block: General questions

Start of Block: Information Sources

Q13 **Information sources**

| 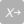 |
| --- |

Q14 Identify if you have received nutrition information, counselling or advice during the last 12 months from any of the people or professions below by tagging one or more options below. (Check all that apply)

- Sports Registered Dietitian/Nutritionist within the Athletic Department or Sport Organization (1)
- Sports Registered Dietitian/Nutritionist outside the Athletic Department or Sport Organization (2)
- Other (3)
- I did not receive any nutrition information, counselling or advice during the last 12 months (4)

| Page Break |  |
| --- | --- |

Display This Question:

If Q14 = Sports Registered Dietitian/Nutritionist within the Athletic Department or Sport Organization

Or Q14 = Sports Registered Dietitian/Nutritionist outside the Athletic Department or Sport Organization

Or Q14 = Other

Carry Forward Selected Choices from "Q14"

| 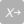 |
| --- |

Q15 Define the number of contact moments that you had with each professional during the last 12 months. (Select an answer for each horizontal row, select NA if non-applicable)

|  | 1-2 visits (1) | 3-6 visits (2) | 7-10 visits (3) | 11 or more visits (4) | NA (5) |
| --- | --- | --- | --- | --- | --- |
| Sports Registered Dietitian/Nutritionist within the Athletic Department or Sport Organization (x2) |  |  |  |  |  |
| Sports Registered Dietitian/Nutritionist outside the Athletic Department or Sport Organization (x3) |  |  |  |  |  |
| Other (x4) |  |  |  |  |  |
| I did not receive any nutrition information, counselling or advice during the last 12 months (x6) |  |  |  |  |  |

| Page Break |  |
| --- | --- |

Display This Question:

If Q14 = Sports Registered Dietitian/Nutritionist within the Athletic Department or Sport Organization

And Q14 = Sports Registered Dietitian/Nutritionist outside the Athletic Department or Sport Organization

And Q14 = Other

| 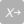 |
| --- |

Q16 Please check all types of information/topics that were addressed in these sessions/materials. (Check all that apply)

- Food first approach (1)
- Risk of nutritional supplement use, especially the ones not provided by the Athletic Department (2)
- Anti-doping education (3)
- Third-party testing (4)
- Ergogenic aids (5)
- Sources that help to make informed decisions on the use of dietary supplements (6)
- Identification of doping related substances that may appear on a product label (7)

| 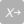 |
| --- |

Q17 Who is your preferred source within the Athletic Department to go to when you have questions about the use of nutritional supplements. (Select only one)

- Sports Dietitian (or sports RD) or dietitian (or RD) (1)
- Athletic Trainer (2)
- Strength & Conditioning Coach (3)
- Coach (4)
- Physician (5)
- Team manager (6)
- Team member (another athlete within your team or Athletic Department) (7)
- Athletic Director (8)

| Page Break |  |
| --- | --- |

| 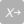 |
| --- |

Q18 What is/are your preferred information source/s when you look for nutritional supplement information? (Check all that apply)

- Scientific literature (i.e., peer-reviewed journal articles) (1)
- Social media sites (2)
- Internet search engines targeting non-scientific websites, such as blogs (3)
- Non-scientific (sport) magazines (4)
- Website of (inter)national anti-doping organization (5)
- Website of your national dietetic organization or internationally recognized websites, such as the IOC, ISSN, and ACSM (6)
- TV or radio ads (7)
- General sales websites, such as Amazon (8)
- Website of sport nutrition or dietary supplement companies (9)
- Website of a nutrition expert (10)
- Podcast (11)
- I do not search for information (12)

End of Block: Information Sources

Start of Block: Social Media

Q19 **Social Media**

| 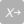 |
| --- |

Q20 Do you use the following types of social media?
(Select a score: No or Yes for all listed items)

|  | No (1) | Yes (2) |
| --- | --- | --- |
| Facebook (1) |  |  |
| Instagram (4) |  |  |
| Snapchat (5) |  |  |
| YouTube (6) |  |  |
| Twitter (7) |  |  |
| TikTok (8) |  |  |
| Pinterest (9) |  |  |
| Other (10) |  |  |

| Page Break |  |
| --- | --- |

Display This Question:

If Q20 [ Yes] (Count) >= 1

Carry Forward Selected Choices from "Q20"

| 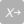 |
| --- |

Q21 How often do you check social media (even if you are logged in all day)?

|  | Not at all (1) | Every few days (2) | Once a day (3) | every few hours (4) | every hour (5) | every 30 minutes (6) | every 10 minutes (7) | every 5 minutes (8) |
| --- | --- | --- | --- | --- | --- | --- | --- | --- |
| Facebook (x1) |  |  |  |  |  |  |  |  |
| Instagram (x4) |  |  |  |  |  |  |  |  |
| Snapchat (x5) |  |  |  |  |  |  |  |  |
| YouTube (x6) |  |  |  |  |  |  |  |  |
| Twitter (x7) |  |  |  |  |  |  |  |  |
| TikTok (x8) |  |  |  |  |  |  |  |  |
| Pinterest (x9) |  |  |  |  |  |  |  |  |
| Other (x10) |  |  |  |  |  |  |  |  |

| Page Break |  |
| --- | --- |

Display This Question:

If Q20 [ Yes] (Count) >= 1

| 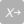 |
| --- |

Q22 How much time do you spend using your combined social media on average per day?

▼ 5 minutes or less (1) ... 10 hours or more (13)

| 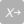 |
| --- |

Q23 How much of your total time on social media do you spend at footage/threads related to nutritional supplements and sports foods? (Select only one)

- Never (1)
- Rarely (2)
- Sometimes (3)
- Often (4)
- A lot (5)

| Page Break |  |
| --- | --- |

| 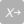 |
| --- |

Q24 What would be the best way for your athletic department to reach you with information related to banned substance education? (Check all that apply)

- In person (onsite) (1)
- In person (online) (2)
- Per email (3)
- Via a central website (4)
- Via an app (5)
- Via Facebook (6)
- Via Twitter (7)
- Via Instagram (8)
- Via TikTok (9)
- Via YouTube (10)
- Via Pinterest (11)
- Via Reddit (12)
- Via LinkedIn (13)

End of Block: Social Media

Start of Block: Supplement Knowledge

Q25 **Nutritional Supplement Knowledge**

| 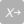 |
| --- |

Q26 The following are statements about athletes' needs for particular micronutrient supplements. (Please select Agree, Disagree, or Not Sure for each item)

|  | Agree (1) | Disagree (2) | Not Sure (3) |
| --- | --- | --- | --- |
| Vitamin C should be routinely supplemented by athletes. (1) |  |  |  |
| B vitamins should be taken when feeling low in energy during exercise. (2) |  |  |  |
| Salt tablets should be used by athletes that get a cramp. (3) |  |  |  |
| Iron tablets should be taken when a player feels extremely tired and is pale (4) |  |  |  |

| Page Break |  |
| --- | --- |

| 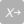 |
| --- |

Q27 The purity and safety of all supplements are tested before sale. (Select only one)

- Agree (1)
- Disagree (2)
- Not Sure (3)

| 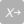 |
| --- |

Q28 Supplement labels may contain false or misleading information. (Select only one)

- Agree (1)
- Disagree (2)
- Not Sure (3)

| Page Break |  |
| --- | --- |

| 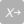 |
| --- |

Q29 The following statements are about the reported benefits of performance-enhancing supplements. (Please select Agree, Disagree, or Not Sure for each item)

|  | Agree (1) | Disagree (2) | Not Sure (3) |
| --- | --- | --- | --- |
| Creatine reduces the perceived effort of exercise by acting on the central nervous system. (1) |  |  |  |
| Caffeine improves the efficiency of muscles at a given rate of oxygen delivery. (2) |  |  |  |
| Beetroot Juice (nitrates) decrease muscle breakdown and reduce muscle soreness (3) |  |  |  |
| Beta-Alanine produces carnosine, a protein that can buffer (“soak up”) acid by-products produced during high intensity activity. (4) |  |  |  |

| Page Break |  |
| --- | --- |

| 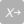 |
| --- |

Q30 In relation to improving sport performance, which of the following supplements do you think has NOT been supported by a strong body of scientific evidence? (Select only one)

- Caffeine (1)
- Ferulic Acid (2)
- Bicarbonate (3)
- Leucine (4)
- Not Sure (5)

| 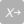 |
| --- |

Q31 Which of the following supplements do you think was banned by the world anti-doping agency (WADA) when exceeding a specific urine threshold but is currently not listed anymore? (Select only one)

- Caffeine (1)
- Bicarbonate (2)
- Carnitine (3)
- Glycerol (4)
- Not Sure (5)

| Page Break |  |
| --- | --- |

| 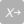 |
| --- |

Q32 Use of contaminated nutritional supplements can lead to a positive doping test. (Select only one)

- Yes (1)
- No (2)
- Not Sure (3)

| 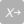 |
| --- |

Q33 Are you familiar with banned substances that may occur in nutritional supplements listed on the WADA (world anti-doping agency) or your own sport specific association? (Select only one)

- Yes (1)
- No (2)
- Not Sure (3)

| Page Break |  |
| --- | --- |

| 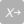 |
| --- |

Q34 I find the idea of using a nutritional supplement that contains banned substances unacceptable. (Select only one)

- Agree (1)
- Disagree (2)
- Not Sure (3)

| 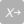 |
| --- |

Q35 Are you aware of the implications within your chosen sport is you were to inadvertently fail a drug test? (select only one)

- Yes (1)
- No (2)

End of Block: Supplement Knowledge

Start of Block: Nutritional supplement use

Q36 **Nutritional Supplement Use**

| 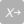 |
| --- |

Q37 At what age did you first use nutritional supplements as an athlete?

▼ Nine years or younger (0) ... 45 (45)

| 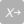 |
| --- |

Q38 Do you purchase or use nutritional supplements outside what is provided by your Athletic Department? (Only select one)

- Yes (1)
- No (2)

| Page Break |  |
| --- | --- |

| 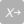 |
| --- |

Q39 What was the frequency of your purchasing third-party tested supplements during the last 12 months? (Only select one)

- Always (1)
- Most of the times (2)
- Sometimes (3)
- Never (4)

| 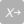 |
| --- |

Q40 If you use supplements or sports foods that your Athletic Department is not providing, who is purchasing them? (Check all that apply)

- I am (1)
- Parents (2)
- Other (3)
- I never use nutritional supplements outside of what the Athletic Department offers me (4)

| Page Break |  |
| --- | --- |

| 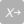 |
| --- |

Q41 Where do you purchase your nutritional supplements? (Check all that apply)

- Brick and mortar store (1)
- Online/ecommerce (2)
- Directly from brand (self-paid) (3)
- Directly from brand (sponsored) (4)
- Indirect via my Athletic Department (self-paid) (5)
- Indirect via my Athletic Department (sponsored) (6)
- I do not purchase supplements (7)

| Page Break |  |
| --- | --- |

| 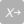 |
| --- |

Q42 Please select the percentage that you think is closest to the proportion of nutritional supplements that have been reported to be contaminated.

▼ 5 (1) ... 100 (20)

| Page Break |  |
| --- | --- |

| 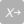 |
| --- |

Q43 Please check all of the nutritional supplements you have used during the last 12 months. (Check all that apply)

- Multivitamin and mineral supplement (1)
- Combination of vitamins (2)
- Combination of minerals (3)
- Single vitamin (for example but not limited to vitamin C, vitamin D, and vitamin E) (4)
- Single mineral (for example but not limited to calcium, iron, magnesium, and zinc) (5)
- Fish oil/ essential fatty acids (6)
- Sports drink (for example but not limited to Powerade, and Gatorade) (7)
- Energy drink (for example but not limited to Red Bull, Monster and other energy drinks) (8)
- Energy gel or chewies (for example but not limited to Gu, Gatorade, and Powerbar) (9)
- Recovery drink (for example but not limited to Muscle Milk, and Rocking Refuel) (10)
- Protein shake (11)
- Weight gainer (12)
- Chocolate milk (or other flavored milk option with sugar added like strawberry milk) (13)
- Sports bar (for example but not limited to an energy bar, and protein bar) (14)
- Energy gel (15)
- Pre-workout supplement (16)
- BCAA (17)
- Leucine (18)
- Beta-Alanine (19)
- Dietary nitrate (for example but not limited to beetroot juice) (20)
- Caffeine (21)
- CLA (22)
- Creatine (23)
- Glucosamine (24)
- Herbs (such as: echinacea, ginseng, and ginkgo biloba or others herbs) (25)
- L-carnitine (26)
- Medium-chain triglycerides (MCT) (27)
- Sodium bicarbonate (28)
- Probiotics (29)
- Ribose (30)
- Quercitine (31)
- Tart Cherry (or other cherry varieties) (32)
- Exotic berries (for example but not limited to acai, and goji) (33)
- HMB (34)
- Ephedra (35)
- Tribulus terrestris (36)
- Maca root powder (37)
- Glycerol (38)
- Colostrum (39)
- CBD (Cannabidiol) (40)
- SARMs (such as: Ostarine, Andarine, Ligandrol (LGD-4033), and RAD140) (41)
- Fenugreek (or also known as: Methi) (42)
- Aswagandha (or also known as: Indian ginseng, poison gooseberry, winter cherry) (43)
- Dendrobium (44)
- Methylliberine (or also known as; dynamine, tetramethylurate or tetramethyluric acid) (45)
- Longjack (46)
- Kava (or also known as: kava kava, awa, ava, yaqona, yagona, seka, malok or malogu) (47)
- Phyllanthus (or leafflower) (48)
- Other (49)
- None of the above (50)

| Page Break |  |
| --- | --- |

Display This Question:

If Q43 = Other

Q44 Please write down any other supplements that you have used in the last 12 months that were not listed just above.

________________________________________________________________

| Page Break |  |
| --- | --- |

Display This Question:

If If Please check all of the nutritional supplements you have used during the last 12 months. (Check a... q://QID116/SelectedChoicesCount Is Greater Than or Equal to 1

And Q43 != None of the above

Carry Forward Selected Choices from "Q43"

| 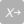 |
| --- |

Q45 Please score each supplement purchased or used during the last 12 months as to whether it was third-party tested.

|  | Yes (1) | No (2) | Not Sure (3) |
| --- | --- | --- | --- |
| Multivitamin and mineral supplement (x1) |  |  |  |
| Combination of vitamins (x4) |  |  |  |
| Combination of minerals (x5) |  |  |  |
| Single vitamin (for example but not limited to vitamin C, vitamin D, and vitamin E) (x6) |  |  |  |
| Single mineral (for example but not limited to calcium, iron, magnesium, and zinc) (x7) |  |  |  |
| Fish oil/ essential fatty acids (x8) |  |  |  |
| Sports drink (for example but not limited to Powerade, and Gatorade) (x3) |  |  |  |
| Energy drink (for example but not limited to Red Bull, Monster and other energy drinks) (x9) |  |  |  |
| Energy gel or chewies (for example but not limited to Gu, Gatorade, and Powerbar) (x10) |  |  |  |
| Recovery drink (for example but not limited to Muscle Milk, and Rocking Refuel) (x11) |  |  |  |
| Protein shake (x12) |  |  |  |
| Weight gainer (x13) |  |  |  |
| Chocolate milk (or other flavored milk option with sugar added like strawberry milk) (x14) |  |  |  |
| Sports bar (for example but not limited to an energy bar, and protein bar) (x15) |  |  |  |
| Energy gel (x16) |  |  |  |
| Pre-workout supplement (x2) |  |  |  |
| BCAA (x17) |  |  |  |
| Leucine (x18) |  |  |  |
| Beta-Alanine (x19) |  |  |  |
| Dietary nitrate (for example but not limited to beetroot juice) (x20) |  |  |  |
| Caffeine (x21) |  |  |  |
| CLA (x22) |  |  |  |
| Creatine (x23) |  |  |  |
| Glucosamine (x24) |  |  |  |
| Herbs (such as: echinacea, ginseng, and ginkgo biloba or others herbs) (x25) |  |  |  |
| L-carnitine (x26) |  |  |  |
| Medium-chain triglycerides (MCT) (x27) |  |  |  |
| Sodium bicarbonate (x28) |  |  |  |
| Probiotics (x29) |  |  |  |
| Ribose (x30) |  |  |  |
| Quercitine (x31) |  |  |  |
| Tart Cherry (or other cherry varieties) (x32) |  |  |  |
| Exotic berries (for example but not limited to acai, and goji) (x33) |  |  |  |
| HMB (x34) |  |  |  |
| Ephedra (x35) |  |  |  |
| Tribulus terrestris (x36) |  |  |  |
| Maca root powder (x37) |  |  |  |
| Glycerol (x38) |  |  |  |
| Colostrum (x39) |  |  |  |
| CBD (Cannabidiol) (x40) |  |  |  |
| SARMs (such as: Ostarine, Andarine, Ligandrol (LGD-4033), and RAD140) (x41) |  |  |  |
| Fenugreek (or also known as: Methi) (x42) |  |  |  |
| Aswagandha (or also known as: Indian ginseng, poison gooseberry, winter cherry) (x43) |  |  |  |
| Dendrobium (x44) |  |  |  |
| Methylliberine (or also known as; dynamine, tetramethylurate or tetramethyluric acid) (x45) |  |  |  |
| Longjack (x46) |  |  |  |
| Kava (or also known as: kava kava, awa, ava, yaqona, yagona, seka, malok or malogu) (x47) |  |  |  |
| Phyllanthus (or leafflower) (x48) |  |  |  |
| Other (x49) |  |  |  |
| None of the above (x50) |  |  |  |

| Page Break |  |
| --- | --- |

| 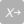 |
| --- |

Q46 Check all boxes of third-party testing systems icons that you recognize from products that you have used during the last 12 months. (Check all that apply)

- Image:Informed choice (1)
- Image:Informed sport (2)
- Image:Nsf certified for sport blue and orange (3)
- Image:Nsf international (4)
- Image:Usp (5)
- Image:Consumer lab logo (6)
- Image:Bscg logo (7)
- Image:Nzvt (8)
- Image:Kolner liste (9)
- I do not recognize any of these icons (10)

End of Block: Nutritional supplement use

Start of Block: Attitude and barriers towards nutritional supplement use

Q47 **Attitude and barriers towards nutritional supplement use**

| 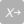 |
| --- |

Q48 I know where to find and order third-party tested supplements. (Only select one)

- Agree (1)
- Disagree (2)
- Not Sure (3)

| 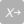 |
| --- |

Q49 I feel that not testing positive is more important than the potential higher price of third-party tested supplements, I feel that there would not be any reason for me not to purchase third-party tested supplements. (Only select one)

- Agree (1)
- Disagree (2)
- Not Sure (3)

| Page Break |  |
| --- | --- |

| 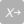 |
| --- |

Q50 I believe that some of the supplements that I want to use are not available as third-party tested supplements. (Only select one)

- Agree (1)
- Disagree (2)
- Not Sure (3)

| 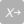 |
| --- |

Q51 I feel that third-party tested supplements are more expensive than untested supplements. (Only select one)

- Agree (1)
- Disagree (2)
- Not Sure (3)

| Page Break |  |
| --- | --- |

| 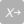 |
| --- |

Q52 I have been using untested supplements and have not tested positive, therefore, there is no need to use third party tested supplements for these products. (Only select one)

- Agree (1)
- Disagree (2)
- Not Sure (3)

| 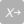 |
| --- |

Q53 If I don’t test positive, the use of each type of supplement improving my performance is allowed. (Only select one)

- Agree (1)
- Disagree (2)
- Not Sure (3)

| Page Break |  |
| --- | --- |

| 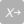 |
| --- |

Q54 A reason to purchase third-party tested supplements is because there is a high risk for contamination of nutritional supplements. (Only select one)

- Agree (1)
- Disagree (2)
- Not Sure (3)

| 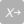 |
| --- |

Q55 The use of third-party tested supplements protects me from testing positive. (Only select one)

- Agree (1)
- Disagree (2)
- Not Sure (3)

| Page Break |  |
| --- | --- |

| 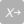 |
| --- |

Q56 When a sport body does not endorse the use of a supplement it limits opportunities for athletes to discuss the use of supplements that are not provided by the Athletic Department. (Only select one)

- Agree (1)
- Disagree (2)
- Not Sure (3)

| 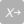 |
| --- |

Q57 I discuss all my supplement choices with the Athletic Departmental Sports RD. (Only select one)

- Agree (1)
- Disagree (2)
- Not Sure (3)

| Page Break |  |
| --- | --- |

| 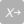 |
| --- |

Q58 If a teammate uses a supplement, I am more likely to try it as well. (Only select one)

- Agree (1)
- Disagree (2)
- Not Sure (3)

| 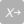 |
| --- |

Q59 A reason not to purchase a third-party tested supplement is because the risk for a nutritional supplement being contaminated is low. (Only select one)

- Agree (1)
- Disagree (2)
- Not Sure (3)

| Page Break |  |
| --- | --- |

| 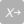 |
| --- |

Q60 It is given that up to 20% of nutritional supplements on the US market contain stimulants and/or banned substances. How can you make sure you do not use contaminated nutritional supplements? (Check all that apply)

- Only use nutritional supplements provided by the Athletic Department (1)
- Only use third-party tested supplements (2)
- Check the label for potentially banned substances (3)
- Research brand integrity before purchase (4)
- My teammates have been using non-third-party tested supplements while not testing positive, which makes it safe for me to use these supplements as well (5)
- Only use what my Athletic Department recommends to be used (6)

| Page Break |  |
| --- | --- |

| 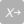 |
| --- |

Q61 The availability of supplements and sports foods within my Athletic Department reduces the need to purchase supplements on my own. (Only select one)

- Agree (1)
- Somewhat Agree (2)
- Not Sure (3)
- Somewhat Disagree (4)
- Disagree (5)

| 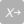 |
| --- |

Q62 I feel that my risk of testing positive would be reduced if I could purchase the supplements that I now purchase on my own via my Athletic Department. (Only select one)

- Agree (1)
- Somewhat Agree (2)
- Not Sure (3)
- Somewhat Disagree (4)
- Disagree (5)

| Page Break |  |
| --- | --- |

| 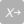 |
| --- |

Q63 I would be okay if my Athletic Department stated that I should not purchase dietary supplements outside of the Department’s currently available product range and I would not purchase on my own supplements that are not so endorsed. (Only select one)

- Agree (1)
- Somewhat Agree (2)
- Not Sure (3)
- Somewhat Disagree (4)
- Disagree (5)

| 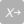 |
| --- |

Q64 I would adhere to not purchasing nutritional supplements without my Athletic Department’s knowledge if the sports RD would state that this is prohibited. (Only select one)

- Agree (1)
- Somewhat Agree (2)
- Not Sure (3)
- Somewhat Disagree (4)
- Disagree (5)

| Page Break |  |
| --- | --- |

| 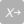 |
| --- |

Q65 I feel that the Sports RD and others within my Athletic Department send out inconsistent signals about the use of dietary supplements. (Only select one)

- Agree (1)
- Somewhat Agree (2)
- Not Sure (3)
- Somewhat Disagree (4)
- Disagree (5)

| 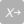 |
| --- |

Q66 I am not comfortable discussing my supplement use with the Sports RD because I already know that the judgment towards my supplement behavior will be negative, potentially resulting in the advice to stop using one or more specific supplements that I like to use. (Only select one)

- Agree (1)
- Somewhat Agree (2)
- Not Sure (3)
- Somewhat Disagree (4)
- Disagree (5)

| Page Break |  |
| --- | --- |

| 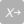 |
| --- |

Q67 I've decided to purchase one or more supplements as a the advice of the sports RD. (Only select one)

- Agree (1)
- Disagree (2)
- Not Sure (3)

| 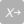 |
| --- |

Q68 I've decided to purchase one or more supplements based on the advice of the physician. (Only select one)

- Agree (1)
- Disagree (2)
- Not Sure (3)

| Page Break |  |
| --- | --- |

| 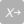 |
| --- |

Q69 I've decided to purchase one or more supplements based on advice commercials adds (on TV, internet, or flyers). (Only select one)

- Agree (1)
- Disagree (2)
- Not Sure (3)

| 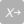 |
| --- |

Q70 I've decided to purchase one or more supplements as a result of social media posts. (Only select one)

- Agree (1)
- Disagree (2)
- Not Sure (3)

| Page Break |  |
| --- | --- |

| 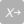 |
| --- |

Q71 I've decided to purchase one or more supplements as a result of the advice of family, friends, or teammates. (Only select one)

- Agree (1)
- Disagree (2)
- Not Sure (3)

| 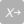 |
| --- |

Q72 I've decided to use one or more supplements based on sponsored contracts. (Only select one)

- Agree (1)
- Disagree (2)
- Not Sure (3)

| Page Break |  |
| --- | --- |

Q73 **Solutions**

Q74 Besides not using nutritional supplements purchased outside of the ones that your Athletic Department provides, the best way to purchase supplements that are not contaminated with doping-related substances is to use third-party tested supplements.

The following propositions aim to gain a better insight into what processes need to be in place to ensure that you will start purchasing or maintain use of third-party tested supplements.

| Page Break |  |
| --- | --- |

| 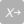 |
| --- |

Q75 My Athletic Department should offer the option of purchasing third-party tested nutritional supplements from them. (Only select one)

- Agree (1)
- Disagree (2)
- Not Sure (3)

| 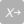 |
| --- |

Q76 If my Athletic Department offers a small selection of evidence-based performance- enhancements (in addition to the existing product offering), I would not buy on my own other products outside the Athletic Department. (Only select one)

- Agree (1)
- Disagree (2)
- Not Sure (3)

| Page Break |  |
| --- | --- |

| 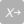 |
| --- |

Q77 While traveling or at home (or not being at the central training location) athletes should have access to third-party tested supplements provided by the Athletic Department. (Only select one)

- Agree (1)
- Disagree (2)
- Not Sure (3)

| 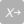 |
| --- |

Q78 Receiving a stipend from the Athletic Department would allow me to purchase third-party tested supplements. (Only select one)

- Agree (1)
- Disagree (2)
- Not Sure (3)

| Page Break |  |
| --- | --- |

| 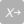 |
| --- |

Q79 I would like to have an app available that allows me to direct order third-party tested supplements. (Only select one)

- Agree (1)
- Disagree (2)
- Not Sure (3)

| 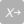 |
| --- |

Q80 What type of information about nutrition supplements and banned substances would you be interested in learning about? (Check all that apply)

- How a product becomes contaminated (1)
- What types of products are usually most “risky” (2)
- Who certifies sport nutrition supplements that are safe to use (3)
- How to find tested products that are safe to use (4)

End of Block: Attitude and barriers towards nutritional supplement use

Start of Block: Personality traits

Q81 **Personality traits**

Q82 The last set of questions is about your personality.

| 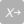 |
| --- |

Q83 It is sometimes hard for me to go on with my work if I am not encouraged. (Only select one)

- True (1)
- False (2)

| Page Break |  |
| --- | --- |

| 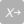 |
| --- |

Q84 I sometimes feel resentful when I don't get my own way. (Only select one)

- True (1)
- False (2)

| 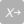 |
| --- |

Q85 On a few occasions, I have given up doing something because I thought too little of my ability. (Only select one)

- True (1)
- False (2)

| Page Break |  |
| --- | --- |

| 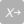 |
| --- |

Q86 There have been times when I felt like rebelling against people in authority even though I knew they were right. (Only select one)

- True (1)
- False (2)

| 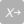 |
| --- |

Q87 No matter who I’m talking to, I’m always a good listener. (Only select one)

- True (1)
- False (2)

| Page Break |  |
| --- | --- |

| 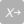 |
| --- |

Q88 There have been occasions when I took advantage of someone. (Only select one)

- True (1)
- False (2)

| 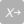 |
| --- |

Q89 I’m always willing to admit it when I make a mistake. (Only select one)

- True (1)
- False (2)

| Page Break |  |
| --- | --- |

| 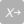 |
| --- |

Q90 I sometimes try to get even, rather than forgive and forget. (Only select one)

- True (1)
- False (2)

| 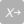 |
| --- |

Q91 I am always courteous, even to people who are disagreeable. (Only select one)

- True (1)
- False (2)

| Page Break |  |
| --- | --- |

| 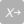 |
| --- |

Q92 I have never been irked when people expressed ideas very different from my own. (Only select one)

- True (1)
- False (2)

| 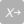 |
| --- |

Q93 There have been times when I was quite jealous of the good fortune of others. (Only select one)

- True (1)
- False (2)

| Page Break |  |
| --- | --- |

| 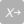 |
| --- |

Q94 I am sometimes irritated by people who ask favors of me. (Only select one)

- True (1)
- False (2)

| 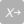 |
| --- |

Q95 I have never deliberately said something that hurt someone’s feelings. (Only select one)

- True (1)
- False (2)

End of Block: Personality traits

Start of Block: End of Questionnaire Incentive

Q96 **Claim your incentive**

Q97 To ensure separate collection of your responses and your personal information you will be directed to another survey after finishing the current questionnaire.

To ensure we only reward athletes that filled out the full questionnaire we want to ask you to fill out a personal code below that you will only know. You will be asked to fill out this code again as part of the next questionnaire. Make sure to write it down before your finish this questionnaire as this code will allow to confirm your submission.

Q98 Please use random words (no names) and select a random number.
Please enter an eight (8) digit pin which includes four (4) letters and four (4) numbers

________________________________________________________________

Q99 After you click the forward button below you will be directed to a separate survey that will ask for your (8) digit pin, first and last name and your email address allowing the research team to provide you with your incentive.

End of Block: End of Questionnaire Incentive
